# Immunological Classification of Tumor Types and Advances in Precision Combination Immunotherapy

**DOI:** 10.3389/fimmu.2022.790113

**Published:** 2022-02-28

**Authors:** Xiufang Ren, Songyi Guo, Xiaojiao Guan, Ye Kang, Jiamei Liu, Xianghong Yang

**Affiliations:** ^1^ Department of Pathology, Shengjing Hospital of China Medical University, Shenyang, China; ^2^ Department of Neurosurgery, The First Hospital of China Medical University, Shenyang, China

**Keywords:** immune, tumor microenvironment, immune score, precision immunotherapy, immunotherapy

## Abstract

Immunity is an important physiological function acquired throughout evolution as a defense system against the invasion of pathogenic microorganisms. The immune system also eliminates senescent cells and maintains homeostasis, monitoring cell mutations and preventing tumor development *via* the action of the immune cells and molecules. Immunotherapy often relies on the interaction of immune cells with the tumor microenvironment (TME). Based on the distribution of the number of lymphocytes (CD3 and CD8) in the center and edge of the tumor and the expression level of B7-H1/PD-L1, tumors are divided into hot tumors, cold tumors, and intermediate tumors (including immune-suppressed and isolated). This review focuses on the advances in precision combination immunotherapy, which has been widely explored in recent years, and its application in different tumor types.

## 1 Introduction

At present, research on the biological characteristics and treatment of tumors has shifted from investigating the tumor itself to investigating the local environment around the tumors and tumor cells, known as the tumor microenvironment (TME). In addition to cancer cells, the TME contains several types of non-cancerous cells, including endothelial cells, pericytes, fibroblasts, and immune cells (1). The TME differs greatly between different types of tumors, different patients with the same tumor type, and even different parts of the same tumor in a patient. This tumor heterogeneity is due to the occurrence of the specific driver and oncogene mutations. The TME has innate immunosuppressive mechanisms and presents adaptive responses to the immune system. The immunosuppressive effect is caused by the genetic changes in the tumor, involving the activation of the WNT-β-catenin, MAPK, JAK, STAT3, and NF-kB signaling pathways, mediating the expression of related cytokines and chemokines to inhibit T cells or the expression of T-cell recruitment factors, and preventing T cells from entering the TME. The adaptive response is triggered by tumor-specific T cells *via* the production of IFN-γ and tumor-infiltrating lymphocytes (TILs), which can induce immune checkpoint molecules (such as CTLA-4) or immunosuppressive mediators (such as IDO1) to exert immunosuppressive effects. Additionally, the TME changes with the progression and recurrence of the disease, complicating the immunotherapy. Tumors develop resistance not only passively as the immune response progresses but also actively by adopting various strategies to interrupt, change, or even terminate anti-tumor immunity. These strategies are collectively referred to as the “immune evasion” mechanism, which often obstructs the inherent development of anti-tumor immunity and results in uncontrollable tumorigenesis.

The basic strategy of anti-tumor immunotherapy is to restore the body’s normal anti-tumor immune response by restarting and maintaining the tumor–immune microcirculation, thereby controlling the growth of and eliminating tumors. This mainly includes monoclonal antibody immune checkpoint inhibitors, therapeutic antibodies, cancer vaccines, and cytokines and small molecule inhibitors. However, all these different approaches have limitations. Over a century, people have mainly studied the enhancement of immune activation. The strategies adopted can generally be divided into two categories: the first method is using effector cells or molecules of the TME to directly attack tumor cells. This is called “passive” immunotherapy, and it utilizes the power of modern technology to achieve higher levels of immunity. It includes targeted therapy of antibodies and their derivatives, such as antibody-drug conjugates (conjugating an antigen expressed on the cancer cell surface) and adoptive cell therapy (genetically modified T-cells, including chimeric antigen receptor T-cell therapy (CAR -T) and T-cell receptor therapy (TCR-T)). The second method is enhancing the immune response through the endogenous regulation and/or activation of immune mechanisms, which is also called “active” immunotherapy. Immune checkpoint therapy is the most representative and revolutionary achievement of “active” immunotherapy. Immune checkpoints are regulators of the immune system that play a critical role in T-cell function, proliferation, and self-tolerance. However, tumors can express inhibitory immune checkpoints and activate diverse immune checkpoint pathways that contribute to immunosuppression in TME. Thus, such molecules can be the targets for immunotherapy. Currently, anti-PD-1 and anti-CTLA4 have been approved by FDA to treat various cancers, and an increasing number of drugs targeting other immune checkpoints (such as TIM3, LAG3, TIGIT, and VISTA) are at the clinical or preclinical stage. However, although such “active” immunotherapy may benefit some patients, it can also push the immune system to a supra-physiological level and lead to an increase in secondary immune-related adverse events (irAEs), which are the unintended effects of the over-activated immune system and can affect almost every organ of the body.

An important clinical observation researchers have made is that systemic immune activation is unnecessary to inhibit cancer growth, especially in solid tumors (1–3). It has been proven that the activation of peripheral tumor-specific T cells is usually unrelated to tumor regression or the prognosis of cancer patients (1). Therefore, it is necessary to formulate specific strategies to correct specific immune deficiencies or dysfunctions in the process of tumor progression and to restore the body’s anti-tumor immunity, which can reduce the damage to normal tissues. These strategies aim to reactivate the suppressed immune response (4, 5). Most patients with advanced cancer do not have immunodeficiency, and their immune system is still generating effector T cells with anti-tumor activity, which is the basis for immunotherapy. PD-1/PD-L1 inhibitors are a treatment strategy adopted when the PD-1/PD-L1 pathway is upregulated. Immune checkpoint inhibitors (ICIs) restore the function of T cells, inhibit T-cell tolerance, and release the existing immune response, thereby preventing newly generated effector T cells from losing their function in the TME (6). Currently, PD-1/PD-L1 inhibitors have been approved by the Food and Drug Administration (FDA) and can be used to effectively treat metastatic melanoma, lung cancer, head and neck cancer, renal cell carcinoma, urothelial cancer, liver cancer, gastric cancer, Hodgkin’s lymphoma, Merkel cell carcinoma, large B-cell lymphoma, cervical cancer, and any microsatellite instability (MSI)-positive tumors (7, 8). The objective response rate of PD-1/PD-L1 inhibitor treatment in multiple tumor types reaches 40%, and one of the factors contributing to this unprecedented success is fewer immune-related side-effects (9).

## 2 Hot Tumors, Cold Tumors, and Intermediate Tumors

**Figure 1:**
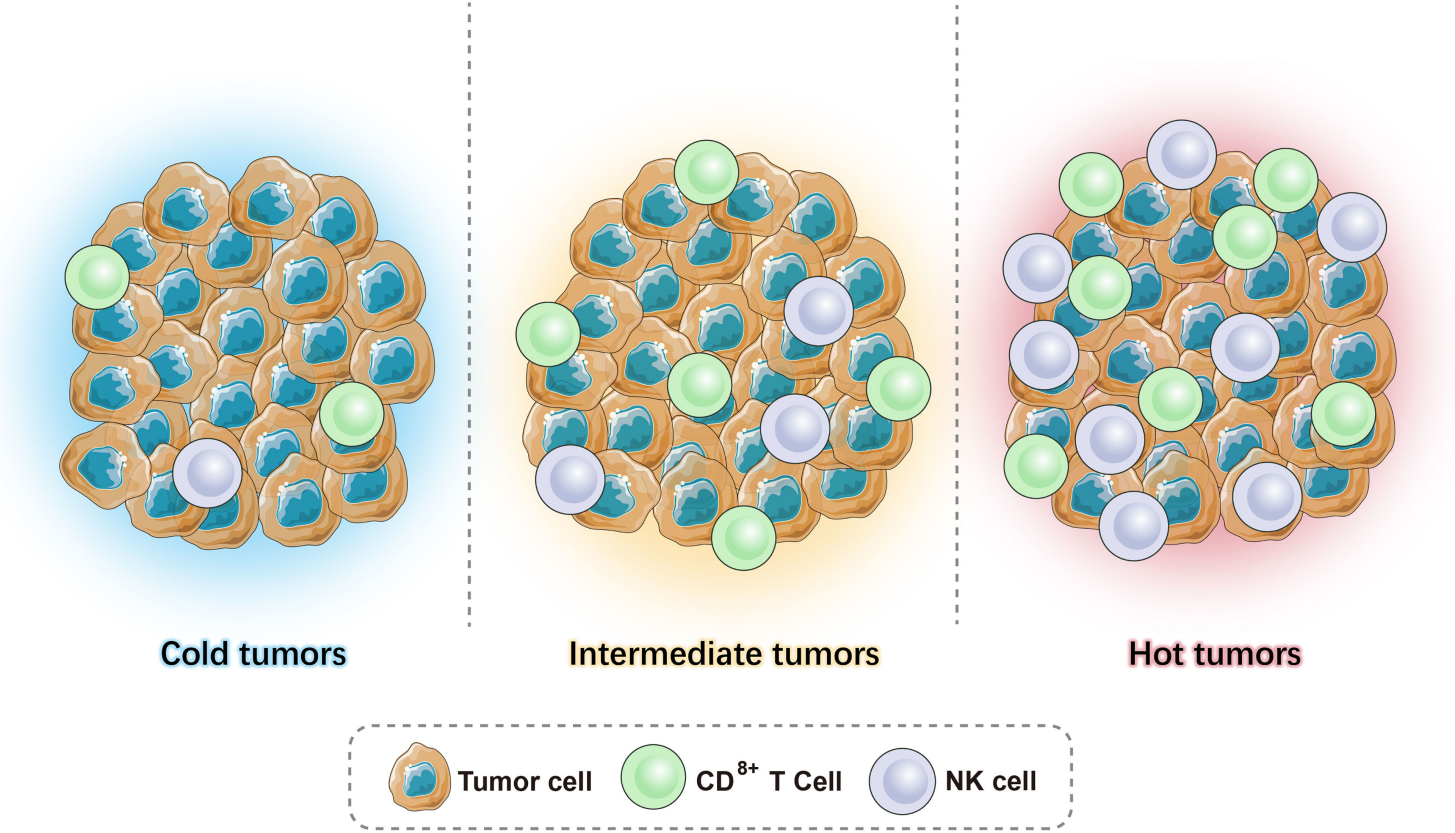
Illustrations of hot tumors, cold tumors, and intermediate tumors.

Given the heterogeneity and interaction between tumors and the immune system, the current tumor classification system cannot meet clinical needs. As T cells are considered to play a major role in fighting cancer, immune scores are particularly important and can help guide clinical treatment. Based on the available literature, we can combine the level of TILs and the expression level of B7-H1/PD-L1 in the TME to classify tumors into (1) high immune score tumor - hot tumor (more infiltrating T cells, high expression of B7-H1/PD-L1); (2) low immune score tumor - cold tumor (almost no T-cell infiltration and expression of B7-H1/PD-L1); (3) medium immune score tumor - intermediate tumor, which can be subdivided into (a) isolated tumor: T cells surround the tumor but do not enter the TME, and (b) immune-suppressed: there is only a small amount of T-cell infiltration in the tumor (**Figure 1**).

The distinction between hot tumors, cold tumors, and intermediate tumors is based on the distribution and number of CD3+ and CD8+ (cytotoxic T cells, CTL) T cells in the tumor. The cytotoxic mechanism of CTLs requires antigen-presenting cells (APCs) to present the antigen to the T-cell surface receptor (TCR) in the form of a major histocompatibility complex I (MHC-I) peptide complex. It is activated with the help of a second signal, the B7-CD28 molecule, which triggers the release of perforin that creates pores in the membranes of target cells (tumor cells), allowing granzymes to enter the cell and degrade the DNA, killing the tumor cells. CTLs secrete IFN-γ, which is an activator of T cells that enhances antigen presentation and surveillance by increasing the expression of immune proteasomes in MHC I and tumor cells. Moreover, it can induce interferon-stimulating genes (ISGs) that impair the proliferation of cancer cells. However, continuous IFN-γ signaling and long-term antigen exposure can lead to T-cell failure and resistance to immune checkpoint blockade, resulting in immunosuppressive effects (10). CD4+ Th1 cells inhibit angiogenesis by inhibiting vascular endothelial growth factor (VEGF) and promote the entry of CD8+ T cells to the TME. In addition to T cells, as APCs, B cells exert a specific immune response *via* the B-cell receptor (BCR) recognition of antigens as they differentiate into plasma cells and eliminate antigens by secreting antibodies (11).

Recently, studies have shown that besides immune cells, these three phenotypes have other important variations in their TME. Hot tumors are associated with a higher expression of interferons (IFN), interleukins, and tumor necrosis factor (TNF), which help T-cell activation and expansion (12). Moreover, a high tumor mutation burden is observed in hot tumors (13, 14). Conversely, cold tumors are characterized by the absence of stimulatory signals and a low expression of neoantigens, which cause poor tumor immunogenicity. The different mechanisms of the three phenotypes give rise to a distinct response to immunotherapy. The current review will discuss and provide a comprehensive view of therapeutic strategies that can be used in combination with immunotherapy, which precisely target these three tumor phenotypes.

## 3 Immunotherapy for Hot Tumors

### 3.1 Immunotherapy Targeting T Cells

The enrichment of T cells in the tumor is the aim of ICI-based monotherapy or combination therapy.

#### 3.1.1 CTLA-4 and PD-1 Therapy

Depleted or dysfunctional TILs express higher levels of inhibitory receptors. The most representative ones are cytotoxic T lymphocyte-associated antigen 4 (CTLA-4) and PD-1 (15, 16). CTLA-4, a homolog of CD28, is highly expressed in Tregs and plays an important role in Treg development and function (17, 18). CTLA-4 inhibits the early activation and differentiation of T cells (usually in the lymph nodes), while PD-1 transmits inhibitory signals and regulates the effector function of T cells (mainly in the tumor) (19). Anti-CTLA-4-PD-1 dual therapy may be effective only in hot and intermediate immunosuppressive tumors as they all rely on T-cell infiltration to some extent (20).

#### 3.1.2 Co-Inhibitory Receptors of T Cells

Lymphocyte activating gene 3 (LAG3) is a co-inhibitory receptor on T cells related to anti-PD-1/PD-L1 therapy and can enhance the activity of Tregs and regulate their proliferation, differentiation, and function (21). The effect of blocking LAG3 as monotherapy is weak; however, it has shown a synergistic effect in preclinical models when used in combination with anti-PD-1. Several clinical trials have also shown that combinatorial therapy targeting TIM-3 and PD-1 can reverse T cell exhaustion and significantly enhance the anti-tumor immune response. Other co-inhibitory receptors, including TIGIT (counteracts the co-stimulatory function of CD226), BTLA (CD272, B/T lymphocyte attenuator), VISTA (T-cell activation V-domain Ig Inhibitor), and SIGLEC9, have been reported to show synergistic anti-tumor effects after co-blockade with PD-1 (13).

#### 3.1.3 Co-Stimulatory Checkpoint Molecules

Co-stimulatory checkpoint molecules, such as CD27, CD28, CD137 (TNFRSF4), and GITR (glucocorticoid-induced TNFR-related protein), promote the expansion and effector functions of T cells when combined with ICIs while controlling the Treg inhibitory function (22). However, such molecules are also highly expressed on Tregs and can promote the anti-inflammatory function of Tregs. Thus, the effect of targeting co-stimulatory molecules is limited and has failed in several clinical trials (12).

### 3.2 Microbial Modulation

Experiments have shown that patients with a “good” gut microbiome (e.g., high *Ruminococcaceae*/*Faecalibacterium* ratio) can mediate and enhance the systemic anti-tumor immune response and improve effector T-cell function in the TME by increasing antigen presentation. On the contrary, patients with a “bad” gut microbiome (low bacterial diversity and high relative cell mass) present limited infiltration of lymphoid and myeloid cells in the tumor, leading to impaired systemic and anti-tumor immune response and weakened antigen presentation ability (23). Recently, several studies showed that it is related to intestinal microbial metabolism. Intestinal *B. pseudolongum* can produce inosine production and enhance the immunotherapy response. Short-chain fatty acids produced by certain gut bacteria can be sensed by DCs and regulatory T cells. Spermidine and vitamin B6 generated by polyamines are capable of stimulating autophagy (24, 25).

**Figure 2:**
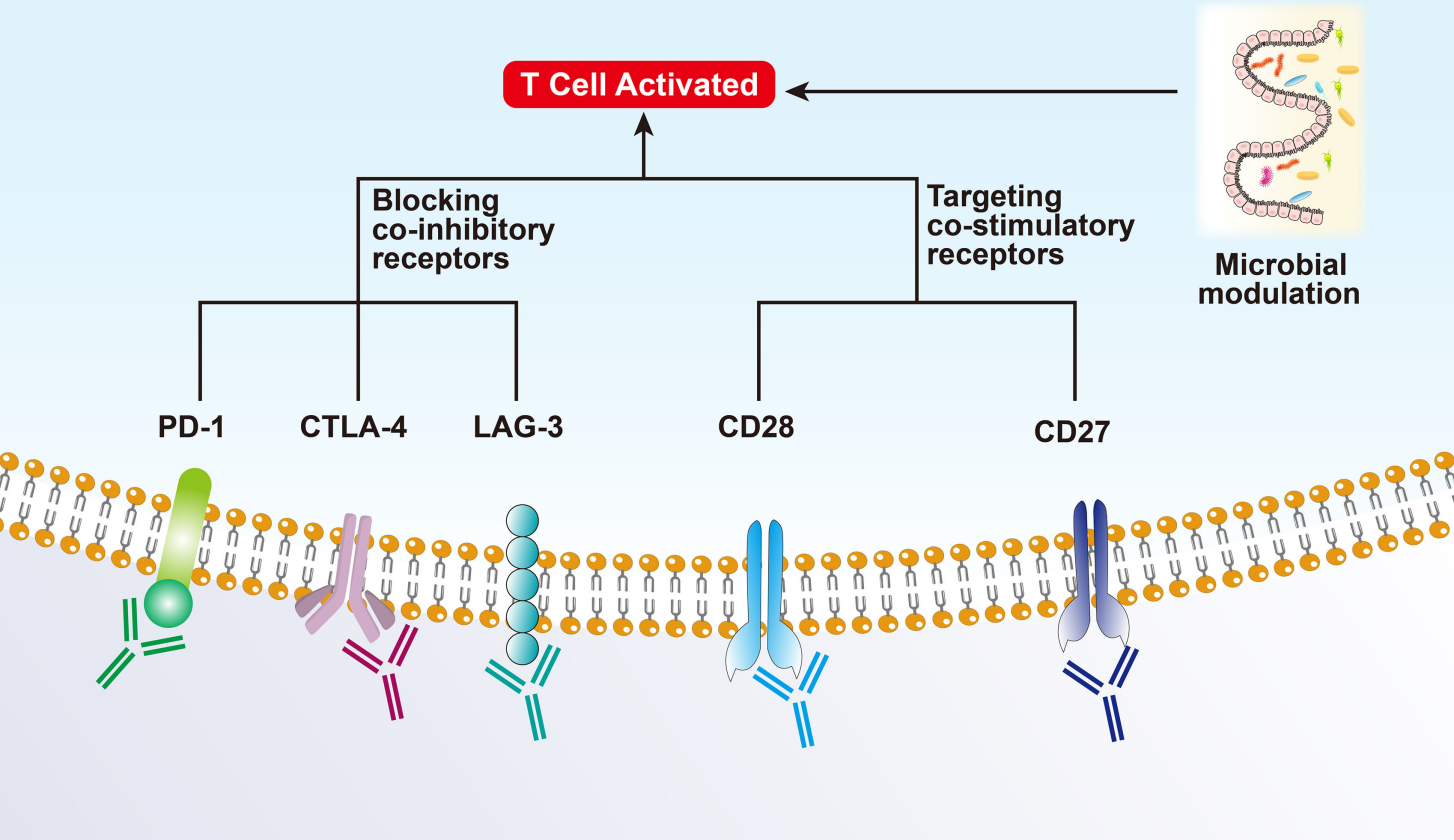
Mechanism of immunotherapy for Hot tumors.

A study on metastatic melanoma showed that there is a significant correlation between symbiotic microbial components and clinical response to PD-1 immunotherapy, highlighting the therapeutic potential of regulating gut microbes in patients receiving ICIs. Nevertheless, only regulating the microbiota may not be able to treat intermediate or cold tumors, it should be combined with other treatments (**Figure 2**).

## 4 Immunotherapy for Intermediate Tumors

### 4.1 T Cell Transport Regulation

In intermediate isolated tumors, as a consequence of genetic and epigenetic changes, which control T-cell recruitment signals (such as T-cell transport chemokines), there is a lower expression of T cells, and CD8+ T cells cannot reach the tumor and distribute around the TME (26). In addition, the activation of the β-catenin pathway can inhibit the recruitment of CD103+ dendritic cells (DC), which in turn affects the effector function of T cells. The activation of the NF-κB/IRF1 axis in cDC1s can also upregulate cytokines and chemokines specialized in the recruitment and activation of cytotoxic T cells (27). TNFSF14 (also known as LIGHT) activates TCR-β receptors and triggers targeted T-cell chemokine production, regulating the recruitment of T cells, and consequently, generating a T-cell-rich microenvironment (26). The epigenetic regulation of the chemokines and blocking of the β-catenin pathway can transform intermediate isolated tumors into hot tumors, thereby increasing the possibility of successful immunotherapy.

### 4.2 Breaking the Physical Barrier

Tumor vasculature is an important physical barrier for T cells due to the imbalance of adhesion molecules, such as intercellular adhesion molecule 1 (ICAM1), vascular cell adhesion protein 1 (VCAM1), and mucosal addressing cell adhesion molecule 1 (MAdCAM1) (28). The changes in the vasculature affect the expression levels of hypoxia-inducible factors (HIFs) leading to a hypoxic environment, which further promotes the formation of an immunosuppressive state in the TME (29). Among HIFs, HIF1α drives the expression of PD-L1 and increases the production of adenosine, which promotes tumor progression. Additionally, the hypoxic environment can affect the proliferation, differentiation, and effector functions of immune cells *via* the secretion of growth factors and cytokines, such as the pro-angiogenic factor VEGF, which can inhibit DC maturation, regulate TCR signal transduction, and inhibit the expression of perforin (PRF1) and granulysin (GNLY), acting against CD4+ Th1 cells and CTL killing tumor cells (30). However, anti-VEGF and drugs targeting other angiogenesis-related factors have failed in treating cancer as single drugs. Experiments have shown that immune checkpoint inhibitors promote the normalization of blood vessels by activating Th1, resulting in improved tumor vascular perfusion, decreased vascular permeability, and reduced hypoxic environment. Therefore, it is possible to combine immune checkpoint inhibitors and anti-angiogenesis therapy to treat intermediate isolated tumors.

### 4.3 Local Adaptation of Immunosuppressive Tumors

Intermediate immunosuppressive tumors have low levels of TIL infiltration, which means that there are no physical barriers or physical barriers that are less obstructive, thereby, enabling the TILs to enter the tumor tissue. Alternatively, the tumor’s immunosuppressive microenvironment could limit the chemotaxis and proliferation of TILs. TAMs and Tregs constitute the two key factors of the immunosuppressive environment. In the hypoxic environment, a large number of cancer-associated fibroblasts (CAFs) and macrophages are re-edited to drive immunosuppression.

There are three major mechanisms that TAMs use to inhibit CTLs directly: (1) by expressing PD-L1 and other immune checkpoint molecules; (2) by producing inhibitory cytokines, such as IL-10 and TGF-β; and (3) by changing the metabolic activity, including metabolite depletion and reactive oxygen species (ROS), among others. In addition, TAMs can indirectly inhibit T-cell function by regulating the immune microenvironment. The main modes include: (1) regulating the structure of blood vessels, and then regulating the extracellular matrix and chemokines to restrict T cells from entering the tumor; and (2) enhancing immunosuppressive cell groups (such as Tregs) or inhibiting the stimulating cells (such as DCs).

CCL2-CCR2 inhibitors can prevent the entry of peripheral monocytes to the tumor inflammation site, resulting in a decrease in the number of TAMs. Although this therapy improved the efficacy of chemotherapy, radiotherapy, and immunotherapy in the preclinical models, it has also been found that blocking CCL-2 and/or CCR2 can induce the release of bone marrow-derived monocytes, which aggravate breast cancer metastasis in mouse models. Blocking CSF1-CSF1R signal transduction can cause TAM apoptosis, especially of the M2 type, leading to the alleviation of the immunosuppressed state of T-cells (31). CSF-CSF1R blockers showed a favorable response after combining with CD40 agonists, PD-L1 immunosuppressants, CTLA-4 antagonists, and adoptive T-cell therapy. Additionally, re-editing TAMs or transforming the M2 to M1 type may be an effective immunotherapeutic method. Re-editing TAMs involves IL-4, IL-13, and immunoglobulin signaling to target and drive the immunosuppressive function. PI3Kγ-targeted therapy drives the immunosuppressive activity of TAMs in multiple pathways including FcγR in lung cancer, pancreatic cancer, and melanoma models (32). In animal models, TAMs can be re-edited by inhibiting PI3Kγ to enhance T-cell immune response.

Tregs are activated by TCR-mediated signals, which inhibit the activation and proliferation of CD4+CD25- and CD8+ T cells, inhibit NK cell proliferation, cytokine secretion, and cytotoxicity, and also inhibit the immune activity of M1 type TAMs, DCs, and B cells. The mechanisms of action of Tregs include: (1) *in vitro*, they act through cell-cell contact-dependent mechanisms, such as CTLA-4, TGF-β, and GITR, which directly bind to the corresponding receptors on target cells, inhibit the expression of IL-2Rα chain, and reduce target cells responsiveness to IL-2, thereby inhibiting the proliferation of effector T cells; (2) *in vivo*, they exert an inhibitory effect *via* inhibitory cytokines IL-10, IL-4, and TGF-β; (3) by declining APC or competitively inhibiting co-stimulatory molecules on APC to inhibit effector T cells; (4) by promoting the expression of granzyme, which kills a variety of autologous target cells *via* the cytotoxic effect of the perforin-dependent mechanism on direct cell contact (33). However, targeting Tregs is very difficult, mainly because of their important role in the autoimmune response and their functions in the normal state. Therefore, clinical treatment cannot be performed using exhaustion methods, and it requires further exploration.

**Figure 3:**
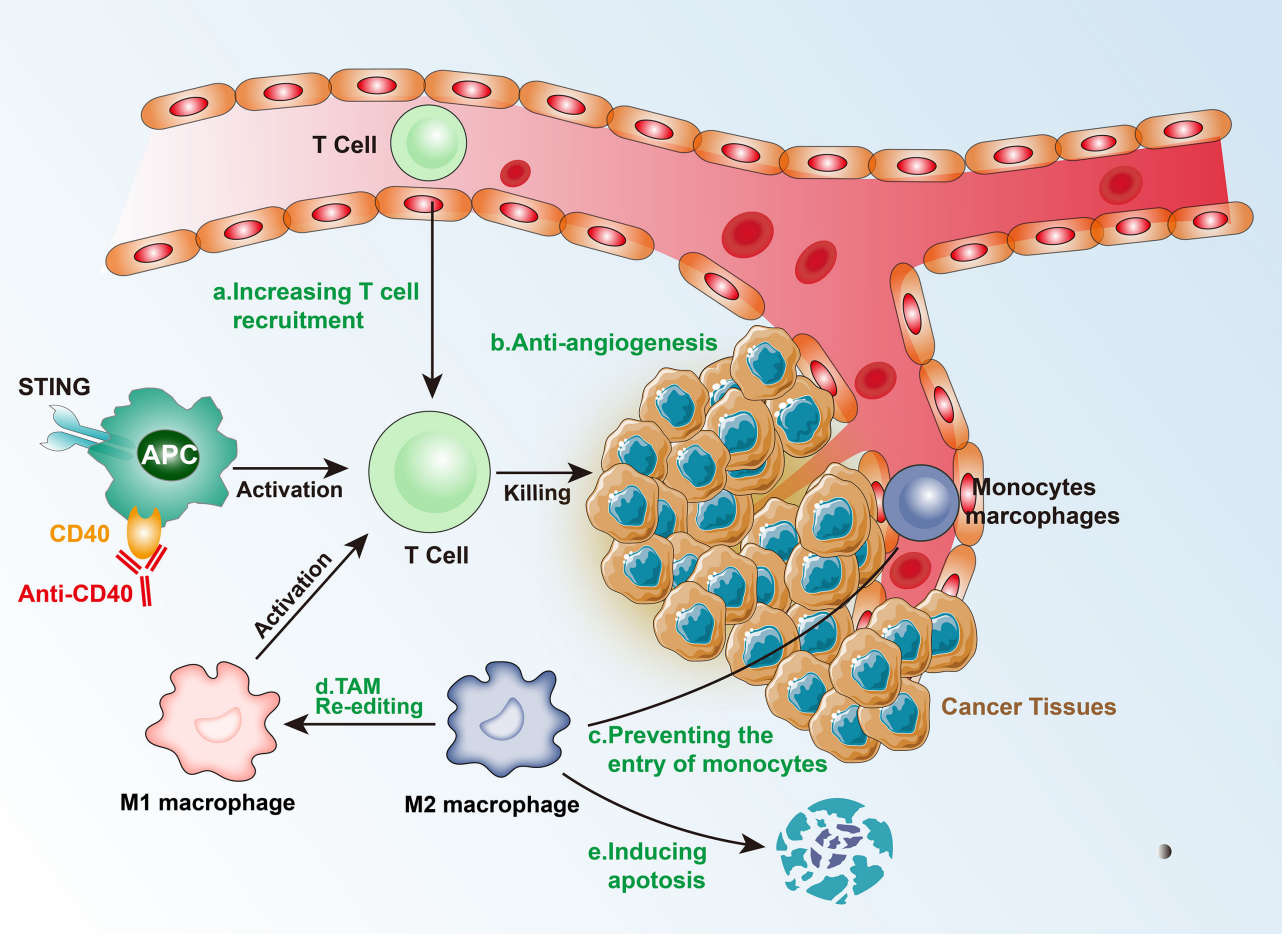
Mechanism of immunotherapy for Intermediate tumors.

There may be an innate immune response defect in immunosuppressive tumors. Injection of interferon gene stimulus (STING) at the tumor site triggers the activation of type I and type II interferons, the increase in DCs in lymph nodes, enhancing the immune response of T cells, and an increase in PD-L1 expression in the TME. Therefore, a subsequent treatment targeting PD-L1 can alleviate the immunosuppressive state and form a TME that is beneficial to immunotherapy (34). However, it is worth noting that continuous administration of STING may also trigger immune resistance. Tumor-infiltrating immune cells (especially APCs) express high levels of toll-like receptors (TLRs) on their surface, which induce the secretion of inflammatory cytokines and the expression of co-stimulatory molecules, and lead to the formation of an immunogenic TME, thereby, inhibiting tumor growth. For APC activation, anti-CD40 monoclonal antibodies can be administered. Therefore, TLR agonists combined with other treatment strategies (such as cancer vaccines) are likely to play an important role in cancer immunotherapy (**Figure 3**).

## 5 Therapy for Cold Tumors

Cold tumors generally have a poor prognosis and are difficult to treat. Cold tumors present the Warburg effect; glycolysis is their leading metabolic pathway, which causes the accumulation of lactic acid and protons in the tumor environment (35). In such tumors, there is almost no oxidative phosphorylation in mitochondria. Moreover, insufficient blood perfusion is a typical feature of solid tumors, especially in high-grade, which accelerates glycolysis, promoting the accumulation of lactic acid in TAMs (36). Tumor-derived lactic acid inhibits the function of T cells and NK cells and the differentiation and activation of myeloid cells; therefore, tumor acidification may be relevant for immune escape. Consequently, treating tumors against the Warburg effect, for example by inhibiting LDH-A, may be an effective strategy. LDH-A-related lactic acid production and acidification impair the expression of IFN-γ on tumor-infiltrating T cells and NK cells, thereby, inhibiting tumor immune monitoring and facilitating tumor growth. However, treatments targeting LDH-A may only reduce lactic acid secretion in certain cancers, and targeted drugs are currently at the pre-clinical stage.

Recent studies have shown that immunogenic cell death (ICD) not only induces tumor cell death but can also promote anti-tumor immune responses *via* the release of tumor neoantigens and damage-associated molecular patterns(DAMPs) (37). These mechanisms can convert cold tumors into immunogenic hot tumors by generating powerful CTL responses that can eliminate tumors (38). The strategies to enhance the immune response in these tumors include tumor vaccines, adoptive cell therapy (ACT), the removal of co-suppressive signals using ICIs or consumption of myeloid-derived suppressor cells (MDSCs), and co-stimulatory signals provided by anti-OX40 or anti-GITR (39). Since T-cell infiltrated hot tumors greatly benefit from anti-tumor T-cell therapy, in general, initiating therapy will be beneficial for cold tumors without T-cell infiltration. However, the coordinated application of multiple immunotherapies may increase the response rate of patients.

### 5.1 Photothermal Therapy (PTT) and Dissolving Microneedles (DMN)

Recently, photothermal therapy (PTT) is becoming popular due to its high selectivity, low treatment resistance, remote controllability, and low systemic toxicity. It functions by converting infrared (NIR) light energy into heat, ablating cancer cells accurately, while resisting tumors release new antigens and DAMPs (40). However, the mild heat produced by PTT promotes the upregulation of IDO, thereby interfering with its downstream effector pathways involved in immunosuppression (41). PTT also induces the secretion of IFN-γ and increases the expression of IDO. High expression of IDO inhibits the activation of APC and reduces antigen presentation; at the same time, IDO can also catalyze the degradation of tryptophan to kynurenine, damaging the activation of CD8+ T cells, and inhibiting their anti-tumor ability by activating regulatory T cells (42, 43). Therefore, administering the PTT-induced tumor vaccine while simultaneously reversing the IDO-mediated immunosuppressive state is a promising strategy in immunotherapy. Compared to systemic administration, the local administration has obvious advantages in superficial tumors, as it can improve bioavailability and avoid off-target effects.

As a new generation of transdermal drug delivery, dissolving microneedles (DMN) can overcome skin barriers and create effective microchannels to deliver the drug targeting the tumor microenvironment (44). Microneedles can be combined with PTT to jointly deliver photosensitizers and IDO blockers to the lesions. This combined strategy shows great therapeutic potential in eradicating primary tumors and preventing distant metastases.

### 5.2 Radiation Therapy

The precise delivery of radiotherapy combined with the resulting ICD may transform tumors into *in-situ* vaccines (45), which can control the tumor itself and possibly control immune system. It was reported that in pancreatic cancer models with low expression of CD8+ T lymphocytes and high expression of PD-L1, there was a poor response to ICIs, vaccines, ICIs + vaccines, and radiotherapy. Zheng et al. proposed a strategy to promote T-cell infiltration in cold tumors (46). Their model proved that the implementation of radiotherapy, vaccines, and ICIs (anti-PD-L1mAb) can decrease tumors and improve patient survival. The success is probably due to radiation, which recruited vaccine-activated T cells, reversing the immunosuppressive environment, and T cells exerting their anti-tumor effects. The combination of radiotherapy and immunotherapy can potentially bring huge clinical benefits.

### 5.3 Chemotherapy

Anti-tumor drugs enhance the immunogenicity of tumor cells primarily *via* antigenicity and adjuvant properties. Genotoxic chemotherapy can induce mutations and trigger the generation of new epitopes, thereby increasing the antigenicity (47). However, these new antigens may be expressed at low levels on the surface of tumor cells and may not cause sufficient immune responses. Anthracyclines, cyclophosphamide, oxaliplatin, and paclitaxel can trigger ICD chemotherapy, which can enhance adjuvant properties by releasing DAMPs and activating apoptosis or necrosis pathways (48). Drugs, such as 5-fluorouracil, can deplete MDSCs in tumors (49), while cyclophosphamide can deplete Tregs and trigger the transfer of immune-stimulating bacteria from the intestinal lumen to the damaged epithelial cells (50). Effector T cells eliminate matrix-mediated chemical resistance in cancer tissues. Therefore, chemotherapy is involved in the positive regulation of the immune system, and because its effectiveness is based on the existing immunity, hot tumors are more likely to benefit from it. Therefore, it is critical to transform cold tumors into hot tumors.

### 5.4 Therapy for Improving the Immunogenicity of Tumor Cells

Insufficient tumor-associated antigen (TAA) load can impair the immune response regulated by effector T cells. It is possible to increase the expression of TAA, promote the re-recruitment of T cells to the tumor site, and enhance tumor cell antigenicity (45). AZA (5-azacytidine) is a cytosine analog, which can effectively inhibit DNA methyltransferase and upregulate MHCIβm, cancer-testis antigens, and related IFN-γ signaling molecules, thereby enhancing the immune response. The analysis of human kinases based on shRNA interference revealed that EGFR, RET kinase, and MEK negatively regulate MHC expression and antigen presentation in several tumors. Therefore, their inhibitors may increase the sensitivity of patients’ immunotherapy, although this method does not seem to be suitable for cold tumors. Spranger et al. conducted a study on patients with melanoma and analyzed hot and cold tumors with no difference in antigen burden and/or mutation burden and proved that lack of the BATF3 lineage of DCs may be essential for “cold” TME (51). Therefore, the assessment of the mutation burden can provide a reasonable basis for the use of therapy that improves the immunogenicity of tumor cells. Additionally, improving the DC function could also be a therapeutic target. Anti-CD40 antibodies have been evaluated in the clinic and found to be tolerable in treating cancer. CD40 mAb upregulate APC function, lead to the activation of T-cell priming, and may be critical in converting cold tumors to hot ones (52).

### 5.5 Targeting the DNA Damage Response

Studies on lung cancer and melanoma have demonstrated that high mutation and neoantigen loads are associated with the clinical benefits of ICIs (53). In addition to the neoantigen load, it is important to inhibit the tumor cell DNA damage response (DDR). For example, the inactivation of *MLH1* inactivates the DNA mismatch repair (MMR) mechanism, causing genome instability, and triggering immune surveillance. ATR serine/threonine, ATM (ataxia vasodilator mutation), checkpoint kinase 1 (CHK1), DNA-dependent protein kinase (DNA-PK), p38MAPK- and MAPK-activated protein kinase 2 (MK2), and other DDR inhibitors can enhance the sensitivity of chemotherapy and radiotherapy and increase the possibility of successful tumor immunotherapy (54).

### 5.6 Adoptive Cell Therapy (ACT)

Since 1988, this strategy relies on culturing patient-derived cancer-specific CD8+ T cells and injecting them back to recognize and kill cancer cells. with the developments in gene-engineering technology, TCR-engineered T cells (TCR-T) and Chimeric Antigen Receptor T-cells (CAR-T) are developed and able to consistently generate cancer-specific T cells by transferring antigen receptors into T cells. FDA has approved CAR-T cells in 2017, and CAR-T cells targeting the CD19 protein are very effective in recognizing and destroying B-cell lymphoma and leukemia cells. However, they can also destroy off-target normal CD19+ B cells in the patient’s body. Besides that, acute cytokine release syndrome (CRS) is another important side-effect of CAR-T, which is the result of the supra-physiological levels of cytokines produced by CAR-T cells after recognizing antigens. All above-mentioned are major limitations of the use of ACT in solid tumors (55). Additionally, TIL therapy seems to be difficult to implement in cold tumors. In a study by Verdegaal et al., two patients with stage IV melanoma were repeatedly stimulated to remove the lesion tissue, establish cell lines, and expand tumor-specific T cells, obtaining favorable results (56). ACT can also be used to analyze the stability of neoantigen-specific T-cell responses against antigens. The selective disappearance of neoantigens recognized by T cells in tumor tissues and the development of neoantigen-specific T cells in TILs constitute a highly dynamic environment. Therefore, it is necessary to find a wide range of neoantigen-specific T cells to avoid tumor tolerance. However, whether ACT treatment can overcome the T cells that cannot be spontaneously activated and turn cold tumors into hot tumors is not known.

### 5.7 Oncolytic Therapy

Oncolytic viruses can infect tumor cells selectively and cause tumor cell lysis. They can also induce the body’s potential anti-tumor immunity effectively and perpetually (57). Moreover, the dying tumor cells can release TAAs and DAMPs, triggering an effective anti-tumor immune response, provided that the oncolytic virus does not contain virulence factors but maintains the ability to stimulate tumors.

T-VEC is a genetically modified herpes simplex virus type 1 (HSV-1) (58) and the first oncolytic anti-viral drug approved by the FDA. The modified T-VEC cannot replicate in normal cells, but it can proliferate in tumor cells and finally lyse them, express the immune activation protein granulocyte-macrophage stimulating factor (GM-CSF), and accelerate the anti-tumor immune response. A phase Ib clinical trial using T-VEC combined with the anti-PD-1 antibody pembrolizumab in patients with advanced melanoma showed a significant remission rate of 62%, with a complete remission rate of 33%. Patients who responded to T-VEC treatment showed an increase in CD8+ T cells, a high mutation load, and high expression of PD-L1 in several cell subgroups of the tumor, indicating that T-VEC followed by anti-PD-1 therapy can effectively decrease tumors that require the presence of specific T cells. Furthermore, a phase II study showed that melanoma patients treated with T-VEC have an increased amount of infiltrating CD8+ T cells in non-injected lesions, indicating T-VEC can induce a systemic immune response and alter the TME (59). This is a potential treatment strategy to “heat” cold tumors.

Aznar et al. (2019) re-used the established yellow fever vaccine 17D with CD137 agonistic antibody for tumor immunotherapy (60). The live attenuated virus strain 17D is used as a preventive vaccine to effectively resist wild-type viruses. Aznar et al. proved that 17D can replicate and kill a variety of tumor cells in humans and mice, qualifying as an oncolytic agent. In addition, they found that the intra-tumor application of the vaccine in a syngeneic mouse model with subcutaneous tumors not only delayed untreated transperitoneal tumors in a CD8 T cell-dependent manner but also delayed the growth of tumors. This observation indicates that the vaccine induces anti-tumor CD8 T cells, which is an important step in the long-term response and success of immune checkpoint modifiers.

### 5.8 Tumor Vaccine

Tumor vaccines stimulate DCs. When tumor antigens appear on the surface of DCs, they not only induce the CTL killing of specific tumor cells but also stimulate cytokine secretion by helper T cells (61). Vaccines are developed using several different methods based on cell, DNA, and protein/peptide preparations. Disappointingly, after thousands of clinical trials of tumor vaccines in a variety of tumor types, only sipuleucel, a tumor vaccine loaded with the prostatic acid phosphatase (PAP) antigen and GM-CSF showed moderate efficacy and was approved by the FDA to treat asymptomatic or mildly symptomatic hormone-refractory prostate cancer. Most tumor vaccines in clinical studies showed only a short time of T-cell response without the ability to maintain the tumor regression effect, which may be due to the immunosuppressive TME. Current research on ICIs emphasizes the clinical benefits of somatic mutations and the emergence of new antigens, and the combined use of ICIs and tumor vaccines in cold tumors may yield promising results. Vaccination therapy combined with subsequent anti-PD-1 therapy has demonstrated efficiency in tumor regression and broadened the durability of vaccine-induced T cell responses in two studies (62, 63).

### 5.9 Cytokines

Cytokines can regulate the expansion, activity, and function of T cells. IL-2 was the first cytokine discovered to promote the growth of T cells, and recombinant IL-2 has shown anti-tumor activity in several mouse tumor models (64). IL-2 was tested in cancer patients and has been approved by the FDA for the treatment of renal cell carcinoma and melanoma, with an objective response rate of 5%–15% (65). However, in some patients, it may cause significant toxicity to organs and tissues, and it is generally related to capillary leak syndrome (66). However, thanks to genetic engineering techniques, IL-2 preferentially binds to CD8 and NK cell IL-2 receptors, refocusing IL-2 into the tumor microenvironment and ameliorating the off-target effects.

**Figure 4:**
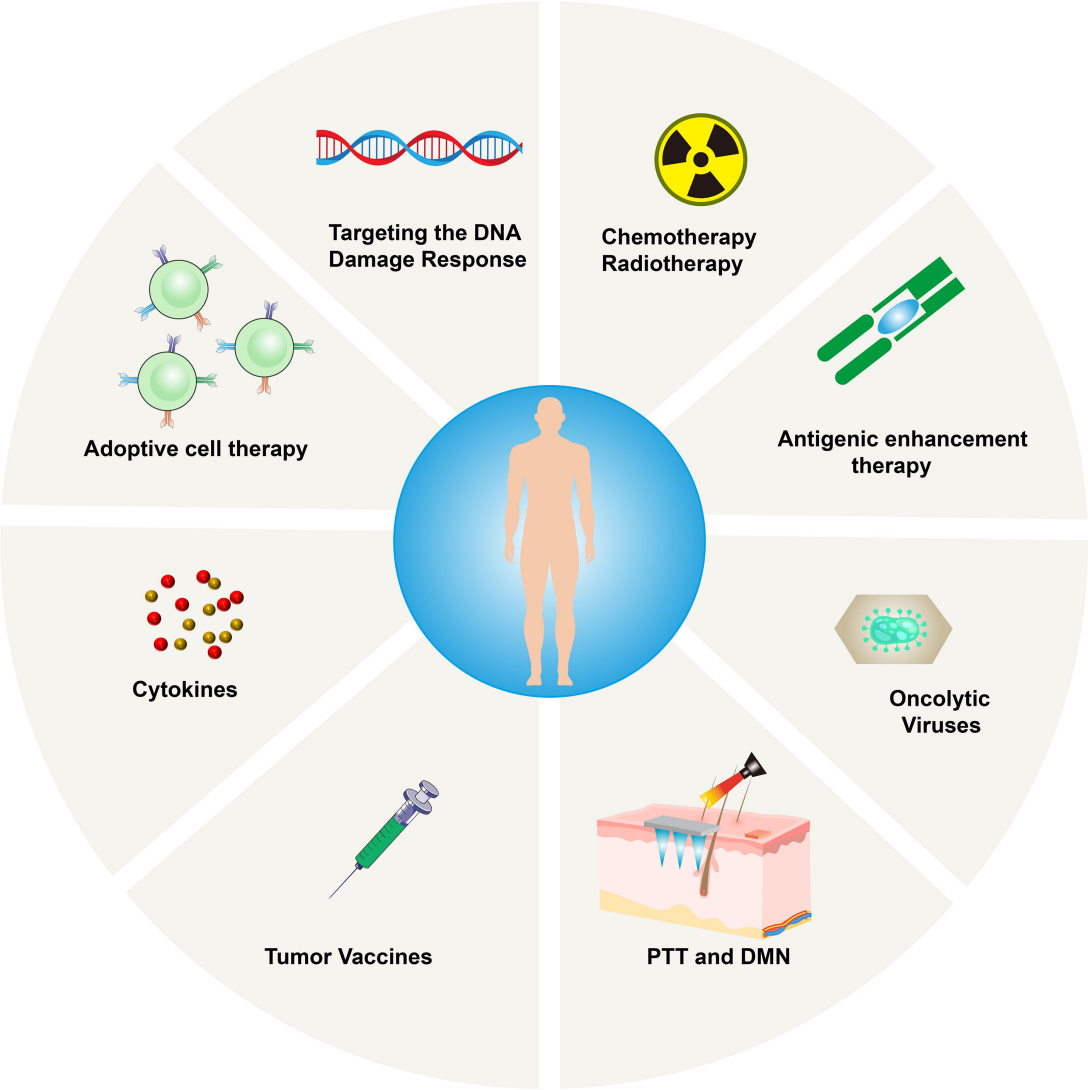
Mechanism of immunotherapy for Cold tumors.

Other cytokines are also being investigated. IL-15, a cytokine related to IL-2, targets NK cells and does not trigger the adverse effects of IL-2. It has, therefore, attracted a lot of attention in tumor immunotherapy recently (67). IL-7 can improve the anti-tumor response of CD8+ T cells and promote their growth, differentiation, and proliferation (68). IL-21 can initiate T-cell activation, counteract the *in-vitro* expression of FOXP3 induced by TGF-β in CD4+ T cells, and alleviate the immune suppression mediated by Tregs (69). IL-12 can enhance the cytotoxicity of CAR-T cells and may be an effective adjuvant for CAR-T cell therapy for Glioblastoma (GBM) (70). IL-21 combined with CXCL13 can also target and regulate TFH (T follicular helper) and B cells in the TME, which are important for the survival of patients (**Figure 4**).

## 6 Conclusion

As scientists and clinicians are gradually realizing the difficulty in the treatment of malignant tumors, it has become necessary to develop novel precise tumor treatments. Compared to other personalized treatment programs guided by genetic testing, precision cellular immunotherapy, as a “living drug”, provides sufficient breadth and feasibility for the rapid development of treatments for specific neoantigenic epitopes. In the current review, we discussed the therapeutic strategies of precision combination immunotherapy to treat hot, intermediate, and cold tumors and identify the most appropriate treatment. The ultimate challenge we face is the lack of a comprehensive understanding of the interactions between the tumor and the immune system and the difficulties in collecting the tumor tissue and analyzing the immune response in the TME. Additionally, on account of tumor heterogeneity, we urgently need to classify tumors accurately, to identify immune and tumor characteristics relevant for precision combination immunotherapy. Therefore, the treatment methods for hot, cold, and intermediate tumors mentioned in the current review have room for development and need to be further investigated in a clinical setting.

## Author Contributions

XR and SG contributed to the conception of the study and write the manuscript. XG participated in the design of the study. YK helped write the manuscript. All authors contributed to the article and approved the submitted version.

## Conflict of Interest

The authors declare that the research was conducted in the absence of any commercial or financial relationships that could be construed as a potential conflict of interest.

## Publisher’s Note

All claims expressed in this article are solely those of the authors and do not necessarily represent those of their affiliated organizations, or those of the publisher, the editors and the reviewers. Any product that may be evaluated in this article, or claim that may be made by its manufacturer, is not guaranteed or endorsed by the publisher.
